# Acceptability and outcomes of an individualized exergaming telePT program for veterans with multiple sclerosis: a pilot study

**DOI:** 10.1186/s40945-020-00089-5

**Published:** 2020-10-01

**Authors:** Shane Chanpimol, Kimberly Benson, Heidi Maloni, Susan Conroy, Mitchell Wallin

**Affiliations:** 1 Neurology Service, Veterans Affairs Medical Center, 50 Irving St. NW, Washington, DC 20422 USA; 2 MS Center of Excellence, Veterans Affairs Medical Center, Washington, DC USA; 3 Physical Medicine & Rehabilitation Service, Veterans Affairs Medical Center, Washington, DC USA; 4 Research Service, VA Maryland Medical System, Baltimore VA Medical Center, Baltimore, USA; 5 Department of Physical Therapy and Rehabilitation Science, University of Maryland School of Medicine, Baltimore, USA

**Keywords:** Telerehabilitation, telePT, Exergaming, Physical therapy, Multiple sclerosis

## Abstract

**Background:**

Physical rehabilitation services are an important component of treatment for persons with multiple sclerosis (PwMS) to improve and maintain physical mobility. However, PwMS often have significant barriers to outpatient physical therapy (PT) services including mobility deficits and lack of transportation. The integration of exercise gaming (exergaming) and telehealth into clinical PT practices may overcome these barriers. The overarching purpose of this pilot study was to evaluate the acceptability and effects of an individualized telePT intervention using exergaming.

**Methods:**

Ten individuals with multiple sclerosis (MS) completed a 12-week exergaming (Jintronix®) telerehabilitation intervention. In order to measure the acceptability of the telerehabilitation intervention, adherence was measured through the tablet-based rehabilitation software and each participant completed a satisfaction questionnaire. Clinical outcome measures were assessed at baseline and post-intervention. To evaluate the efficacy of this intervention, the following measures of physical function and fatigue were included; the Short Physical Performance Battery (SPPB), 25-Foot Walk (25FW), Modified Fatigue Impact Scale (MFIS), Multiple Sclerosis Walking Scale-12 (MSWS), and the 2-Minute Walk Test (2MWT). Clinical outcomes were analyzed using the Sign test and Wilcoxon signed rank test. All other data were evaluated using descriptive statistics.

**Results:**

After the intervention, participants demonstrated significant improvements in ambulation speed during the 25FW (*p =* 0.04) and ambulation distance during the 2MWT (*p* = 0.002). Statistically significant increases of SPPB total score (*p* = .04) and sub-scores were also found. Participants did not demonstrate significant changes in the MFIS (*p* = 0.31) or MSWS-12 (*p* = 0.06) after the intervention. Participants had a 58.3% adherence rate during the intervention and performed their exercise program an average of 2.5 times per week. All participants reported that they were either ‘satisfied or ‘very satisfied’ with their telerehabilitation experience, would use telerehabilitation again, and would recommend telerehabilitation to others.

**Conclusion:**

This individualized telerehabilitation intervention which integrates exergaming and clinical video teleconferencing is acceptable to patients and may offer a viable alternative to traditional PT for PwMS.

**Trial registration:**

NCT03655431, retrospectively registered on August 31st, 2018.

## Background

Multiple sclerosis (MS) is a progressive debilitating disease of the central nervous system which may cause deficits of physical mobility, balance, and endurance. Physical rehabilitation is prescribed to address existing impairments and minimize the progressive loss of physical mobility, independence, and quality of life over the course of the disease [1]. Physical therapy (PT) interventions are an integral part of physical rehabilitation. PT has been shown to stabilize or improve many physical symptoms of MS including loss of strength [2], balance dysfunction [3], impaired mobility [4], and fatigue [5]. These benefits have led many practitioners to consider physical exercise as a non-pharmaceutical disease modifying treatment [6]. However, PT services are frequently underutilized due to poor access to specialty MS care, geographic accessibility, physical mobility, and cost [7, 8]. In a survey of 1065 persons with MS (PwMS), 21% reported unmet PT needs [7]. The same study also reported that PwMS living in urban or suburban areas were significantly more likely to have accessed PT services compared to those in rural or small-town settings [7].

TelePT, the remote provision of PT services, is improving access to care by using technologies such as clinical video teleconferencing (CVT) and web-based exercise prescription [9]. These services have been shown to reduce burdens of access by decreasing traveling distance, traveling time, and cost for patients and providers [10–12]. There is also growing evidence that telePT for patients with neurological disorders can provide similar outcomes compared to in-person PT [13, 14]. The clinical use of technology is not only improving accessibility of PT services, but also transforming how patients engage in therapeutic exercise [15]. The use of exercise gaming (exergaming) and virtual reality in the clinic and home setting has been shown to provide physical benefits for neurological patients [16–19]. However, the remote provision and monitoring of therapeutic exergames for neurorehabilitation has not been widely studied.

Recent reviews of exergaming have demonstrated the efficacy of balance and gait training for individuals with stroke [16, 20, 21], Parkinson’s disease [17, 22], and MS [18]. The enjoyment of exergames has also been noted as a tool to improve adherence to exercise programs [20, 22, 23]. Majority of exergaming interventions utilize the Nintendo Wii system or Xbox Kinect system games (e.g. – Wii Sports, Kinect Adventures) [24]. These games often lack the ability to independently control the intensity and volume for each activity due to their commercial nature and healthy target demographic [23]. Overall, this can negatively impact the ability of a therapist to individualize and optimally progress therapeutic exergaming prescription. However, exergaming platforms such as the Jintronix Rehabilitation system allow the therapist to remotely monitor the patient’s performance for each prescribed exergaming activity. Additionally, each activity can be progressed by the therapist remotely using quantitative data recorded by the system such as repetitions, duration of activity, and scores. This affords the therapist greater control and progression of exergaming activities to address individual physical impairments compared to commonly used commercial gaming systems [23]. Therefore, telePT interventions that utilize engaging and highly customizable exergaming programs can be a valuable alternative to in-person therapies for PwMS that have significant burdens to access.

The purpose of this pilot study was to evaluate the acceptability and physical effects of an individualized exergaming-enhanced telePT intervention. This study evaluated acceptability by assessing exercise adherence and satisfaction. Physical outcomes, specifically, mobility and fatigue were also evaluated before and after the intervention.

## Methods

### Participants

Ten individuals with MS were enrolled in the study. Persons with MS referred to PT services for gait or balance dysfunction from the Washington DC Veteran Affairs Medical Center were assessed for participation in the telePT protocol. To be included, subjects must have had a clinical diagnosis of MS based on the McDonald criteria [25], and Expanded Disability Status Scale (EDSS) score of 3.0–6.5 [26]. A Screen of cognitive ability (Montreal Cognitive Assessment score > 23/30) to independently operate the tablet-based exercise software was also required [27]. In addition, participants were required to demonstrate difficulty for attending in-person appointments. Participants either lived greater than 40 miles from the medical facility or could not independently transport themselves due to mobility limitations to meet this criterion. Subjects were ineligible to participate if they had unstable cardiopulmonary conditions or experienced an MS-related exacerbation within the previous 3 months of starting the intervention. This study was approved by the Washington DC Veteran Affairs Internal Review Board and Research and Development Committee.

### Measures

Acceptability of the intervention was assessed by participant adherence and satisfaction. Participants were instructed to perform their exercise programs 3 or more times per week. Adherence to the prescribed exercise program was recorded by the Jintronix rehabilitation software and monitored remotely by the therapist via a secure web-based clinical portal. Participant satisfaction and personal cost-savings were assessed by a post-intervention questionnaire.

Measures of physical ability and fatigue were collected to assess the efficacy of this telePT intervention. Baseline measures were taken during an initial in-person visit prior to starting the telePT intervention. Post-intervention measures were taken during an in-person visit within one week after the participant’s last telePT follow-up visit. Participants completed the Short Physical Performance Battery (SPPB), 25-Foot Walk (25FW), Modified Fatigue Impact Scale (MFIS), Multiple Sclerosis Walking Scale-12 (MSWS-12), and 2-Minute Walk Test (2MWT) before and after the intervention. The SPPB [28] assesses standing balance, walking speed, and a timed five time sit-to-stand test. The rating scale starts at “0” indicating no performance or low performance to a score of “4” indicating high performance with a total range of 0 to 12. The 25FW [29] assesses the time (seconds) it takes the participant to walk 25 ft as fast and as safely as possible. The 25FW is the average of 2 trials that are performed consecutively. If necessary, assistive devices can be used. The MFIS [30] is a subjective report of fatigue consisting of 21 items with scoring ranges between 0 and 82, a higher score reflecting greater fatigue impact on daily activities. The MSWS-12 [31] is a questionnaire which measures self-reported limitations of walking due to MS during the past 2 weeks. Each item ranges from 1 to 5 with a total score range of 0 to 60, and higher scores indicate greater degree of limitation. The 2MWT [32] measures ambulatory function and endurance by having the participant walk as far as they can in 2 min.

### Equipment

This intervention utilized an Xbox Kinect sensor and a Dell Latitude 115,175 tablet configured by GovSphere, Inc. (www.govsphere.com) for security and compatibility within the Veteran Affairs Intranet. Each tablet contained an exercise software called VITAL Rehab, produced by Jintronix, Inc. (www.jintronix.com), which was utilized to provide the telePT intervention. Jintronix software version 2.0.2 was utilized for the intervention. Jintronix also provides a web-based clinical portal for therapists to monitor exercise performance and quality. Therapists can adjust the participants home exercise program (HEP) in the same application. Each tablet supported CVT for live video follow-ups. Tablets were WIFI enabled and could also utilize cellular 3G data if participants did not have their own high-speed internet service. Technical support was provided by GovSphere, Inc. as needed. Common technical issues addressed were getting devices connected to home WIFI and ensuring proper placement of Kinect camera to perform motion-capture.

### Intervention

**Fig. 1:**
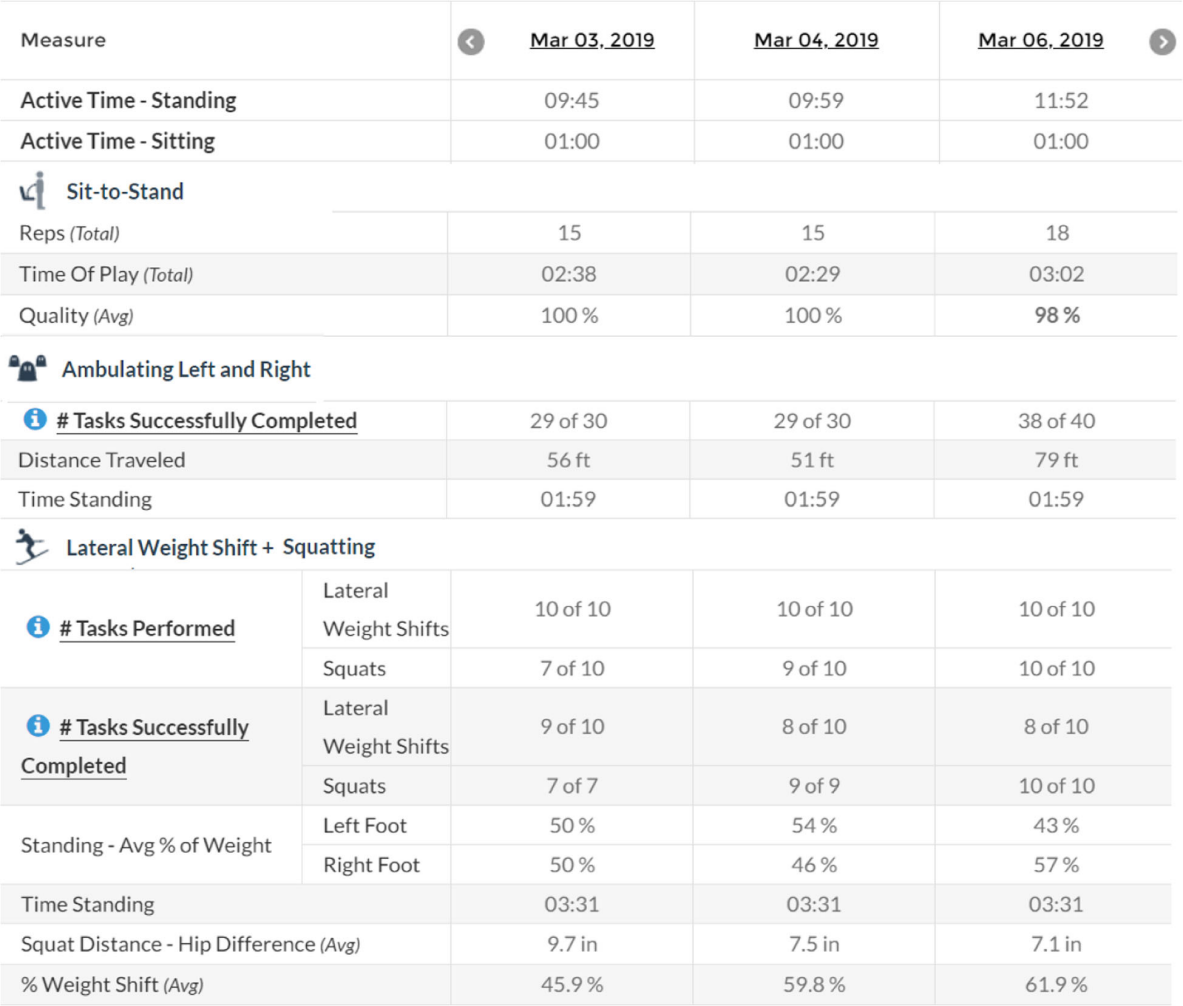
Example of Jintronix web portal report of exercise performance. Parameters such as repetitions, duration of activity, and tasks completed successfully were closely monitored when progressing activities. Adherence was calculated using the login date and time

**Fig. 2:**
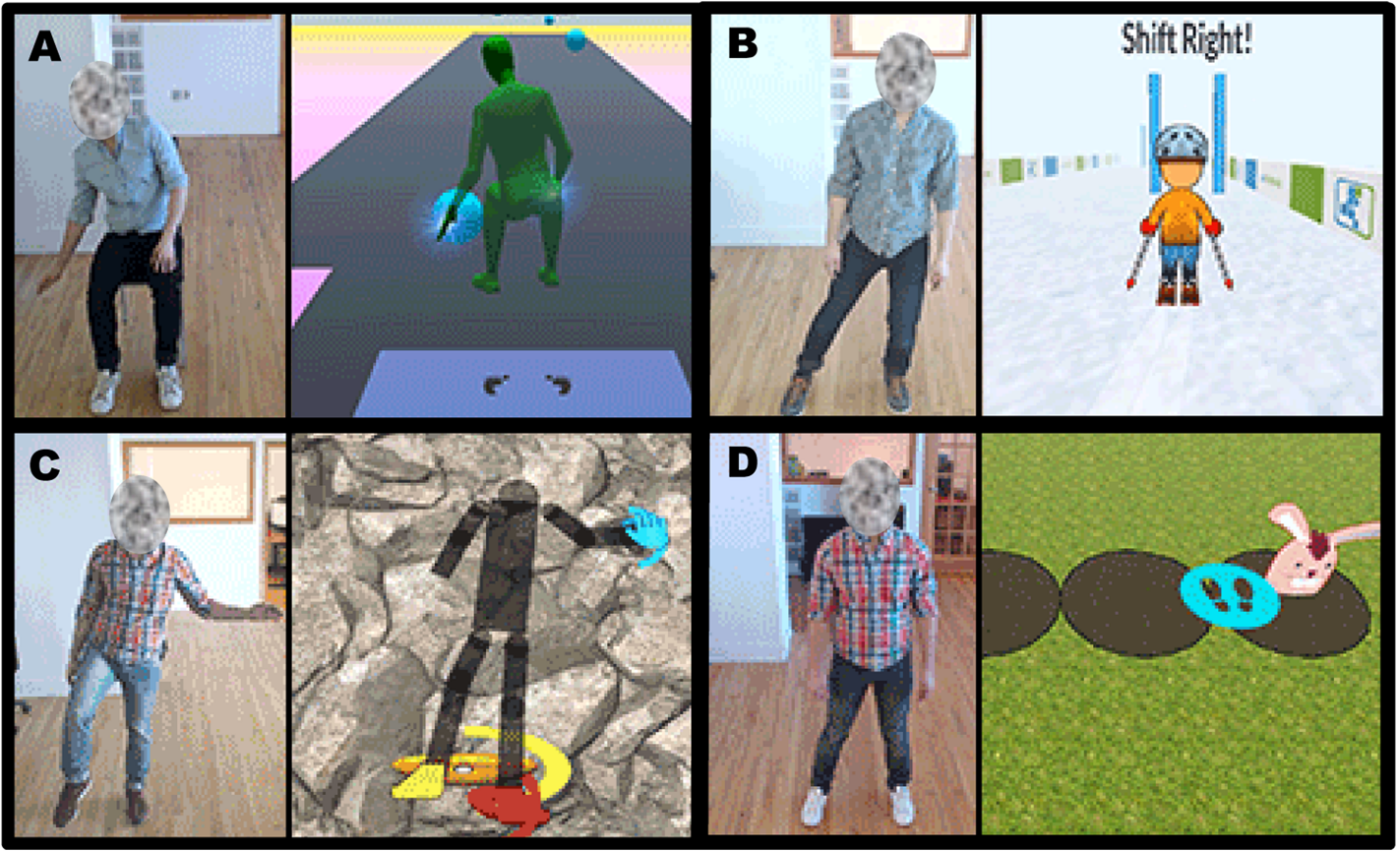
Example Jintronix exergames include; **a** – Popping balloons: reaching in all directions in sitting position, **b** – Skiing: lateral weight shifting in standing, **c** – Rock climb: single leg stance and lateral reach, **d** –Rabbit Bop: sidestepping

Patients completed an in-person physical therapy assessment and were oriented to the tablet and exercise software. Participants were scheduled to follow-up with the PT once a week via CVT for 12 weeks. In the event of technical issues, CVT appointments were rescheduled or held via telephone conference. Each participant’s HEP was developed by the PT using the Jintronix exercise library to address limitations identified during the initial assessment. The Jintronix exercise library included an array of exergame options for varying functional levels. Exergames could be completed in sitting or standing postures and included activities which focused on active range of motion, strength, balance, or calisthenic movements. Each activity required the participant to complete a goal-directed task or replicate movements of an on-screen avatar. Therapists developed each participant’s HEP to take approximately 30 min to complete. Performance reports, as shown in Fig. 1, were available to monitor adherence, accuracy, repetitions completed, and total activity time. Therapists could utilize these reports and participant feedback to adjust the challenge of exergames remotely. For example, static and dynamic balance activities could be adjusted to further challenge reaction time, coordination, amplitude of weight shift, and duration in unstable postures. Repetitions, sets, and duration parameters could be controlled for strength and endurance activities. Examples of exergames prescribed are included in Fig. 2.

### Statistical methods

All statistics were analyzed using R Studio i386 3.5.0 for Windows. Demographic data, adherence, and satisfaction were evaluated using descriptive statistics. The SPPB, MFIS, and MSWS-12 data which are measured on the ordinal scale were analyzed using a two-tailed Sign test. The 25FW and 2MWT data which are measured on the continuous scale were analyzed using a two-tailed Wilcoxon signed rank test. No a-priori assumptions were made, and statistical significance was determined using a critical α of 0.05.

## Results

Ten individuals with MS participated in the telePT program. Participant demographic and clinical characteristics are presented in Table 1. Majority of the participants were female (80%) and presented with RRMS (90%). Half of the participants utilized an AD for indoor ambulation and demonstrated a median EDSS of 5.0.

**Table 1:** Demographics and Clinical Presentation

**Age (yrs.)**	49.6 (9.0)
**Gender**	8 F, 2 M
**MS Type**	
RR	9 (90%)
SP	1 (10%)
**Years Since Dx**	8.0 (6.3)
**EDSS**	5.0 [3.5–6.0]
**Commute**^a^	
Miles	40.9 (36.0)
Minutes	77.8 (35.3)
**Indoor Assistive Device Use**	5 (50%)

^a^ Indicates one-way to medical facility. Note: Values are presented as mean (SD) or median [range]. *Dx* diagnosis, *mi* miles, *min* minutes, *EDSS* Expanded Disability Status Scale, *AD* assistant device, *PT* physical therapy, *RR* relapsing remitting MS, *SP* secondary progressive MS, *RW* rolling walker, *SPC* single point cane, *BD* balance dysfunction, *GD* gait dysfunction

**Fig. 3:**
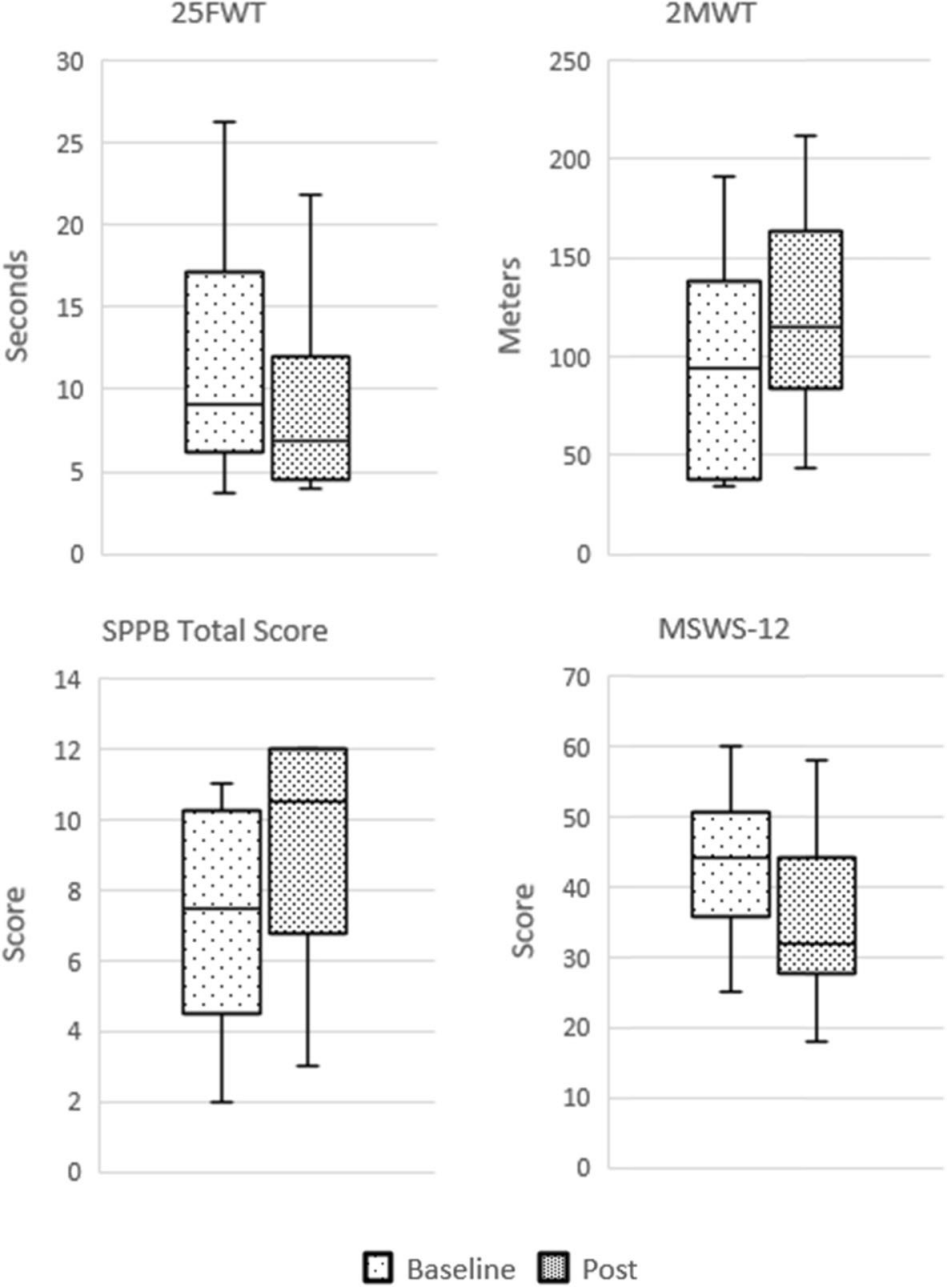
Gait and mobility outcomes:25FW, 25-ft walk; SPPB, Short Physical Performance Battery; 2MWT, 2-min walk test; MSWS-12, Multiple Sclerosis Walking Scale

Table 2 demonstrates average baseline, post-intervention, and differences as well as *p*-values for the 25FW and 2MWT which were analyzed using the Wilcoxon signed rank test. Table 3 shows median scores and p-values for the SPPB, MFIS, and MSWS-12 which were analyzed using the Wilcoxon sign test. After the intervention, participants demonstrated a mean increase of 24.4 ± 12.6 m (*p* = .002) during the 2MWT. Participants also improved their gait speed by 2.9 ± 4.1 s (*p* = .03) on the 25FW. Participants showed a significant positive median score difference in the SPPB total score (*p* = .002). The balance (*p =* .05), gait (*p =* .05), and chair stand (*p =* .002) subscales also demonstrated positive median score differences. Participants reported an average decrease of 4.3 ± 11.4 points on the MFIS total score though no significant difference of the median scores was observed (*p =* .32) Similarly, no significant median difference was noted in the physical (*p =* .32), cognitive (*p =* .06), or psychosocial (*p =* .26) subscales of the MFIS. Participants did not demonstrate a significant median score difference on the MSWS-12 (*p* = .06) though reported an average reduction of 8.5 ± 9.9 points. Figure 3 depicts gait and mobility outcomes.

**Table 2:** Mean Intervention Outcomes

	Score, mean (SD)			
Measure	Baseline	Post-intervention	Change	***p***-value
25FW (sec)	11.8 (7.4)	8.9 (5.6)	−2.9 (4.1)	.037
2MWT (m)	97.8 (53.0)	122.2 (50.8)	24.4 (12.6)	.002
MFIS				
Physical	23.8 (8.9)	22.3 (8.4)	−1.5 (6.4)	–
Cognitive	21.7 (8.9)	17.8 (9.5)	−3.9 (5.6)	–
Psychosocial	4.0 (2.4)	4.8 (2.6)	0.8 (1.8)	–
Total	49.2 (16.5)	44.9 (17.8)	−4.3 (11.4)	–
MSWS-12	43.3 (10.0)	34.8 (12.0)	−8.5 (9.9)	–
SPPB				
Balance	2.8 (1.2)	3.4 (1.1)	0.6 (0.8)	–
Gait	2.8 (1.1)	3.2 (1.1)	0.4 (0.5)	–
Chair Stand	1.7 (1.4)	2.8 (1.4)	1.1 (1.0)	–
Total	7.3 (3.2)	9.4 (3.2)	2.1 (1.4)	–

See Table 3 for *p*-values of measures which analyzed median scores. *SD* standard deviation, *25FW* 25-ft walk test, *2MWT* 2-min walk test, *MFIS* Modified Fatigue Impact Scale, *MSWS-12* Multiple Sclerosis Walking Scale, *SPPB* Short Physical Performance Battery

**Table 3:** Median Intervention Outcomes

	Score, median [IQR]			
Measure	Baseline	Post-intervention	Change	***p***-value
25FW (sec)	9.1 [6.4–15.0]	6.9 [4.9–10.9]	−1.8 [− 4.1–0.1]	–
2MWT (m)	94.8 [49.1–127.4]	114.8 [94.3–153.5]	23.8 [19.0–28.8]	–
MFIS				
Physical	25.5 [25.0–28.5]	22.5 [18.5–26.0]	−2.5 [− 6.3–1.5]	.317
Cognitive	21.0 [14.0–28.3]	16.0 [11.3–22.3]	−4.0 [− 7.5 – − 1.3]	.058
Psychosocial	4.0 [2.3–5.0]	4.5 [3.3–7.3]	0.5 [0.0–1.8]	.256
Total	53.0 [48.3–59.5]	45.0 [37.3–49.8]	−6.0 [− 11.0–1.5]	.317
MSWS-12	44.0 [37–49.0]	32 [29.3–39.8]	−10.5 [− 13.0 – − 2.5]	.057
SPPB				
Balance	3.0 [2.0–4.0]	4.0 [3.3–4.0]	0.0 [0.0–1.0]	.046
Gait	3.5 [1.3–4.0]	4.0 [2.3–4.0]	0.0 [0.0–1.0]	.046
Chair Stand	1.5 [1.0–2.8]	3.5 [1.3–4.0]	1.0 [0.3–1.8]	.008
Total	7.5 [5.5–9.8]	10.5 [7.5–12.0]	2.0 [1.3–2.8]	.002

*IQR* interquartile range, *25FW* 25-ft walk test, *2MWT* 2-min walk test, *MFIS* Modified Fatigue Impact Scale, *MSWS-12* Multiple Sclerosis Walking Scale, *SPPB* Short Physical Performance Battery

No adverse events occurred during the intervention. Table 4 reports adherence metrics during the intervention. Through the telerehabilitation intervention, participants completed an average of 9.8 ± 2.7 CVT follow-up appointments and needed to reschedule or cancel their appointment an average of 2.2 ± 2.1 times. On average, participants exercised a total of 32.4 ± 10.6 times with a frequency of 2.5 ± 0.8 times per week. During each month of intervention, patients exercised 2.6 ± 1.1 times, 2.6 ± 0.8 times, and 2.3 ± 1.1 times, respectively. Overall, participants were adherent to exercise frequency recommendations an average of 7.0 ± 3.3 weeks of the total 12-week intervention.

**Table 4:** Adherence Outcomes

	Mean (SD)
Total CVT Appointments	9.8 (2.7)
Total RS/CA	2.2 (2.1)
Exercise by week	
Weeks 1–4	2.6 (1.1)
Weeks 5–8	2.6 (0.8)
Weeks 9–12	2.3 (1.1)
Exercised per week	2.5 (0.8)
Exercise total	32.4 (10.6)
Weeks exercised ≥3	7.0 (3.3)

*SD* standard deviation, *CVT* clinical video telehealth, *RS* reschedule, *CA* cancellation, *HEP* home exercise program

Based on satisfaction questionnaire responses, shown in Table 5, all participants reported that they were either ‘satisfied’ (10%) or ‘very satisfied’ (90%) with their telerehabilitation experience. All participants also reported that they would use telerehabilitation again and would recommend telerehabilitation to others. Finally, participants reported cost-savings related to gas (70%), hotels (20%), meals (20%) and lost work (20%). Most participants reported total personal cost-savings between $101 and $500 (60%).

**Table 5:** Satisfaction survey

Question	n (%)
How satisfied were you with your telerehabilitation experience?	
Very Satisfied	9 (90%)
Satisfied	1 (10%)
Somewhat Satisfied	0 (0%)
Not Satisfied	0 (0%)
Would you use telerehabilitation again to receive medical care in the future?	10 (100%)
Would you recommend telerehabilitation to other Veterans?	10 (100%)
Did you experience any cost-savings using telerehabilitation?	9 (90%)
If so, which expenses were reduced?	
Gas	7 (70%)
Hotel	2 (20%)
Meals	1 (10%)
Lost work time	2 (20%)
Other	0 (0%)
What was your total estimated cost savings using telerehabilitation compared to traditional (in-person) care?	
$0–$100	2 (20%)
$101–$300	3 (30%)
$301–$500	3 (30%)
$501–$1000	1 (10%)
> $1000	1 (10%)

## Discussion

### Summary

This pilot study demonstrated that an individualized exergaming telePT intervention using the Jintronix Rehabilitation system was acceptable and may offer positive physical benefits. High levels of adherence to exercise recommendations and reported satisfaction suggest that the exergame telerehabilitation intervention was well accepted. In addition, this intervention demonstrated promising clinical outcomes as patients showed improvements of gait and mobility. These results hold promise that exergaming telerehabilitation may be a clinically beneficial and convenient alternative to traditional outpatient PT.

### Acceptability

On average, participants adhered to exercise recommendations for 7.0 weeks or 58.3% of the intervention period. However, participants exercised 2.5 times per week over the total 12-week intervention which corresponds to 83% of the weekly recommended dosage. A recent systematic review of HEP adherence interventions reported adherence rates ranging from 33 to 93% with an average of 67% adherence [33]. Paul et al. also described similar adherence levels (40 to 63%) to a web-based HEP intervention in PwMS [34]. These adherence levels suggest that the telePT intervention was acceptable and comparable to other in-person and telerehabilitation HEP interventions. Several other web-based exercise telerehabilitation studies note waning adherence in the later stages of the intervention period [34–36]. Adherence in the current study demonstrated only a minimal decrease as the intervention progressed. The relatively steady adherence throughout the intervention may have occurred due to increased engagement from exergames and weekly CVT follow-up with patients.

Post-intervention questionnaires also indicated that the telerehabilitation protocol was well received. All participants were either very satisfied or satisfied with their telerehabilitation experience and reported cost savings related to reduced travel burden. On average, participants would have had to travel an average of 81.8 miles and 2.6 h to maintain a single outpatient PT appointment each week. Participants kept an average of 9.8 of the scheduled CVT appointments. Therefore, this telerehabilitation intervention effectively eliminated an average of 801.6 miles and 25.5 h of travel per participant over the course of the study.

### Mobility and gait

Participants with lower levels of disability (EDSS 3.0–5.0) primarily focused on challenging dynamic balance activities such as side stepping, stepping in all directions, and single-leg stance. Participants with higher levels of disability (EDSS 5.5–6.0) focused on progressing static balance activities such as lateral weight shifting and reaching for virtual objects. These participants were then transitioned to dynamic balance activities once safe and appropriate. All participants performed lower extremity strengthening activities such as repeated sit-to-stands or standing hip abduction. The significant improvements of the 25FW, 2MWT, and the SPPB imply endurance and physical function improved as a result. The average gait speed during the 25FW decreased by 2.9 s or 24.6%. This improvement surpasses the established minimally clinical important difference of 17–20% [37]. The average 2MWT increased by 24.4 m which exceeds the minimal detectable change of 19.21 m [38]. The average improvement of 1.6 points on the SPPB also exceeded the established minimally clinical important difference of 1.0 points for community-dwelling older adults [39]. These findings are further supported by the reduction of self-reported ambulatory limitations on the MSWS-12 which trended toward significance.

Exergame and telerehabilitation studies in PwMS and other neurological conditions support these findings. Exergaming interventions using the Kinect sensor have been shown to improve balance and physical mobility for individuals after stroke [40, 41], traumatic brain injury [42, 43], spinal cord injury [44], and with Parkinson’s disease [17]. A recent review of exergaming interventions in PwMS noted improvements of balance and gait outcomes across numerous studies [23]. The reviewed studies predominantly utilized the Nintendo Wii. Only one randomized controlled study using the Kinect sensor was noted. This study by Ortiz-Gutiérrez et al. utilized a CVT supervised Kinect-based intervention which demonstrated significant improvements of balance function in PwMS [45]. Telerehabilitation models which do not incorporate exergaming technology have also been shown to improve physical outcomes [46]. Finkelstein et al. [36], and Frevel et al. [47] have reported similar findings of increased balance and mobility using web-based HEP interventions in PwMS.

Since majority of exergaming interventions have utilized commercial games in the past, there are particularly few studies which examine the combined effects of exergaming with remote patient monitoring. The ability to monitor exercise performance and progress HEPs remotely is a key differentiation of the current study compared to other exergaming and telerehabilitation interventions. Compared to the use of commercially available exergames, this capability likely allows for more responsive and rapid progression of exercises [23]. Feasibility of exergaming interventions which utilize remote monitoring and progression have been demonstrated in other neurological populations such as stroke [48, 49] and Parkinson’s disease [50]. However, to the authors’ knowledge no studies have yet to evaluate these same intervention parameters for PwMS.

### Fatigue

Despite notable physical improvements participants did not report changes of fatigue based on the MFIS. The total score decreased 4.3 points or 8.7% which falls well short of the minimal detectable change of 19.3% [51]. A meta-analysis of therapeutic exercise interventions which assessed fatigue in MS demonstrated very low to moderate levels of evidence to improve fatigue based on the type of intervention [5]. The current telePT intervention primarily utilized lower extremity strengthening and balance exergaming activities. Therefore, it would be classified as muscle power training or ‘other’ interventions which both demonstrated a low level of evidence to improve fatigue in PwMS.

The highest level of evidence for the improvement of fatigue in PwMS was demonstrated for exercise interventions which incorporated cardiovascular endurance training [5]. Exergames have been shown to elicit moderate intensity cardiovascular training in healthy populations [52, 53]. However, there is conflicting evidence whether exergames provide the same intensity for individuals with neurological deficits, particularly while playing commercial games designed for healthy individuals [23, 54]. Since majority of individualized needs for subjects in the current study required lower extremity strengthening and balance training it is unlikely that exergames exceeded light intensity training. Future exergaming studies should consider monitoring exercise intensity via rate of perceived exertion or heart rate monitoring to maximize potential improvements of fatigue in PwMS.

### Limitations

There are limitations which should be considered related to this pilot study and its methodology. First, conclusions drawn from statistical tests should be taken with caution due to the low number of subjects. As pilot work, the trend and direction of outcomes should hold greatest emphasis during interpretation. Minimal detectable changes and minimally clinically important changes were provided for additional context regarding amplitude of effect. Second, the lack of a control group did not allow comparison to traditional therapy or no therapy. Third, inclusion in the study was notably influenced by travel burden rather than specific physical function deficits solely which may limit generalizability to individuals with MS who are referred for PT services. Lastly, no follow-up period was included. Therefore, it is unknown if and for how long the observed benefits were maintained. The feasibility and acceptability established in the current protocol will guide protocol design and development to address these limitations in future studies.

## Conclusion

Telerehabilitation which combines Kinect-based exergaming, remote patient monitoring, and CVT follow-up is acceptable and shows promise as a potential alternative to in-person outpatient therapies to improve ambulation and mobility outcomes in veterans with MS. The potential physical benefits are further complemented by reduced barriers to access and reduced travel burden to maintain appointments. This and other telerehabilitation interventions warrant further research in larger randomized trials.

## Data Availability

The data is owned by the US Department of Veterans Affairs and can be made available upon request.

## Abbreviations

MS: Multiple sclerosis
PT: Physical therapy
PwMS: Persons with multiple sclerosis
CVT: Clinical video telehealth
VR: Virtual reality
EDSS: Expanded Disability Status Scale
HEP: Home-exercise program
SPPB: Short physical performance battery
25FWT: 25-ft walk test
MFIS: Modified fatigue impact scale
MSWS-12: Multiple sclerosis walking scale
2MWT: 2-min walk test
